# Association of weight-adjusted-waist index with asthma prevalence and the age of first asthma onset in United States adults

**DOI:** 10.3389/fendo.2023.1116621

**Published:** 2023-02-20

**Authors:** Longshan Yu, Yan Chen, Ming Xu, Rongfu Li, Juan Zhang, Shouwei Zhu, Zongbao He, Mingwei Chen, Gaosheng Wang

**Affiliations:** ^1^ Department of Emergency Medicine: The First Affiliated Hospital of USTC, Division of Life Sciences and Medicine, University of Science and Technology of China, Hefei, Anhui, China; ^2^ Department of General Practice, Wuhu City Second People`s Hospital, Wuhu, Anhu, China; ^3^ Department of Endocrinology, the First Affiliated Hospital of Anhui Medical University, Hefei, Anhui, China

**Keywords:** asthma prevalence, WWI index, metabolic syndrome, age at first onset of asthma, cross-sectional studies

## Abstract

**Objective:**

The objective of this study was to assess whether the weight-adjusted-waist index(WWI) is associated with the prevalence of asthma and age when first asthma onset appears in US adults.

**Methods:**

For analysis we selected participants from the National Health and Nutrition Examination Survey(NHANES)database between 2001 and 2018. A dose-response curve was calculated using logistic regression,subgroup analysis,and a dose-response curve.

**Results:**

The study included 44480 people over the age of 20,including 6061 reported with asthma, and the increase in asthma prevalence was 15% associated with each unit increase in the WWI, after adjusting for all confounders(odds ratio(OR)=1.15,95% CI:1.11,1.20). The sensitivity analysis was performed by trichotomizing the WWI, and compared to the lowest tertile, the highest tertile WWI group displayed a 29% increase in asthma prevalence(OR=1.29,95% CI:1.19,1.40). A nonlinear correlation was found between the WWI index and the risk of asthma onset, with a threshold saturation effect indicating an inflection point of 10.53 (log-likelihood ratio test, P<0.05), as well as a positive linear correlation with age at first asthma onset.

**Conclusions:**

A higher WWI index was associated with an increased prevalence of asthma and an older age of first asthma onset.

## Introduction

Asthma is a common chronic respiratory disease. Exacerbations are inevitable for asthma patients,even following medical guidance, resulting in further decline in lung function (1). In the past few decades, asthma prevalence has steadily increased.According to the 2008-2010 Global Burden of Disease(GBD)study, there are 334 million people worldwide suffer from asthma (2). Asthma led to 1.6 million hospitalizations and 183,000 emergency department visits in 2017 (3). In 2009, deaths due to asthma per 10,000 people with asthma were 1.9 in adults and 0.3 in children (4). A large portion of the direct medical costs of asthma are related to hospitalization for severe or poorly controlled asthma (5). As a result,asthma has become one of the most common diseases worldwide,resulting in a significant burden on society (6). By identifying its risk factors,including smoking, alcohol consumption, air pollution, and occupational exposures, asthma can be prevented (6–8).

There is an increase in asthma prevalence due to unhealthy dietary patterns becoming more prevalent. According to research, obese people in the United States are responsible for 250,000 asthma cases a year (9). Adolescents with obesity and overweight subjects to increased risk of asthma (10). The metabolic complications associated with obesity are not the same for everyone. Traditionally, body mass index(BMI)is used to assess obesity, but it cannot differentiate between lean body mass and fat mass (11). The presence of visceral adiposity in conjunction with central obesity can be more relevant to poor metabolic characteristics and is increasingly valued by researchers (12). Furthermore, numerous studies indicate that visceral adipose tissue is more closely associated with diabetes, hypertension, cardiovascular disease and cardiometabolic risk factors than subcutaneous adipose tissue (13–15). In order to assess body fat amount and distribution, a variety of methods are used, including densitometry(dual-energy X-ray absorptiometry, DXA), magnetic resonance imaging(MRI), computed tomography(CT), and mechanical methods. These methods are characterized with high accuracy in assessing body fat,and the first three provide fat imaging and location (16). Since these processes are technically complex, time-consuming and high cost, they cannot be routinely used in clinical settings.The weight-adjusted waist index(WWI) was proposed (17) in 2018. Comparing to BMI, the WWI is a better indicator of fat and muscle mass composition, and it primarily reflects central obesity, independent of body weight (18). Among adults with increased WWI, there was an increased prevalence of hypertension (19), proteinuria (11), cardiovascular mortality (20), and hyperuricemia (21). Nevertheless, no studies have been conducted to determine, if WWI is associated with asthma prevalence. Our objective of this research is to determine the value of the WWI in estimating asthma prevalence in United States(US)adults.

## Materials and methods

### Study design and participants

**Figure 1:**
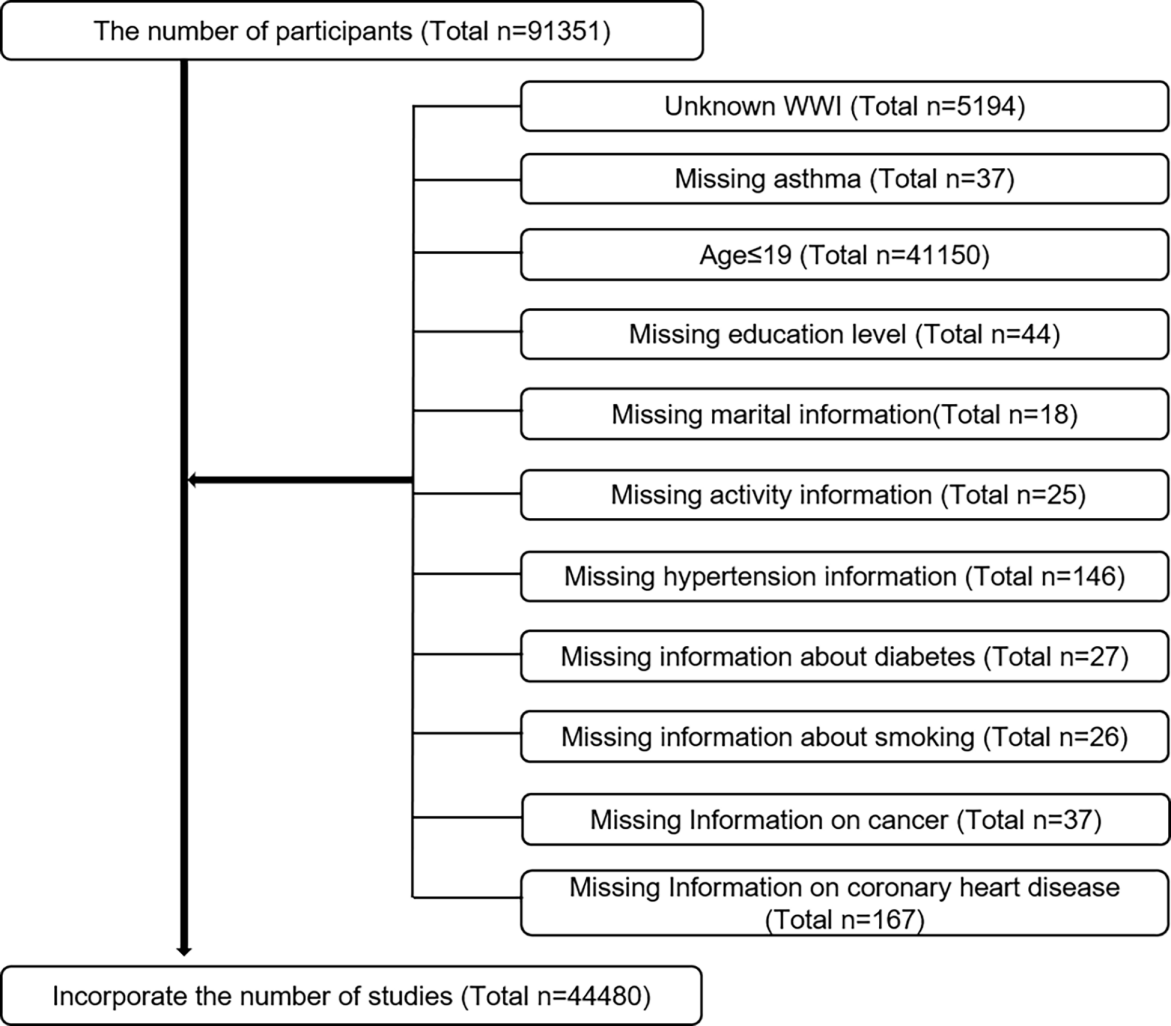
The participants selecting flow chart.

Using baseline clinical data from the National Health and Nutrition Examination Survey(NHANES) from 2001 to 2018, the Centers for Disease Control and Prevention(CDC) monitored US population health every other year using cross-sectional survey methods. A written consent form was submitted by every participant during the NHANES study, which was approved and reviewed by the National Center for Health Statistics Institutional Review Board (NCHS). Surveys were conducted over nine consecutive two-year periods and asthma questionnaires were included in the evaluation. The age at which participants first developed asthma was recorded for those who explicitly answered whether they had asthma and whether it was their first time. Participants in the survey totaled 91,351. The following exclusion criteria were used (**Figure 1**). Finally a total of 44,480 cases were included in this study, including 6061 self-reported ones.

### Collection and definition of data

As an exposure variable,the WWI index was designed. The WWI for each participant was calculated as WC in centimeters divided by the square root of weight in kilograms and then rounded to two decimal places. To measure asthma, questionnaires were used, including:

- “Have you ever been told that you have asthma?”
- “What was your age when you first had asthma?”

The occurrence of asthma and age at first asthma onset were designed as outcome variables.

The multivariate adjusted models summarized potential relationship between the WWI index and asthma. Covariates in our study includes:

- gender(male/female),
- age(years),
- race,
- education level,
- poverty to income ratio(PIR),
- marital status(married or living with partner/single),
- alcohol consumption(drinking or not),
- physical activity(vigorous/moderate/never),
- cholesterol level(mg/dl),
- fasting glucose(mg/dl),
- serumrum uracid(mg/dl),
- smoking status(smoking or not),
- hypertension(or not),
- diabetes mellitus(or not),
- coronary heart disease(yes/no),
- cancer(yes/no),
- blood relatives with asthma,
- dietary intake factors including energy intake,
- fat intake,
- sugar intake,
- water intake.

Besides, the average consumption of the two 24-hour dietary recalls was used for the analysis of all participants.

When continuous variables had a large number of missing values, we converted them into categorical variables. Details of the measurement procedures using the study variables are available at http://www.cdc.gov/nchs/nhanes/s/.

### Statistical methods

A complete statistical analysis of the NHANES data was conducted with three types of sampling weights, stratifications, and clusterings to illustrate the manner of selected participant.Sample weights reflecting selection and response probabilities were used to generate unbiased national estimates. New sampling weights for the combined survey cycle were constructed by dividing the 2-year weights for each cycle by 6 according to the NHANES analysis guidelines. The survey design R package was used to interpret the complex multistage stratified sampling technique of NHANES using the weights provided by the dataset. Continuous variables were represented with weighted survey means and 95% confidence intervals, and categorical variables were represented with weighted survey means and 95% confidence intervals. To exclude cointegration, we used the cointegration test. A sample with VIF greater than 5 was considered to have a cointegration problem. We used multiple logistic regression models to study the relationship between the WWI index, different trichotomies of the WWI index,and asthma prevalence based on the guidelines (22). In model 1, no covariates were adjusted for. In model 2, gender, age, race, marital status and education level adjustment was applied. In model 3, all the variables listed above was adjusted. Smoothed curve fitting(penalized spline method) and generalized additive model (GAM) regression were performed to further evaluatethe relationship between the WWI index and asthma prevalence. Inflection point values were obtained by a likelihood ratio test when a nonlinear relationship was determined to exist. Multiple regression analyses were performed stratified by sex, age, race, hypertension, diabetes mellitus and presence of blood relatives with asthma. P<0.05 was considered statistically significant. All analyses were performed using Empower software. www.empowerstats.com(X&Y Solutions, Inc., Boston, Massachusetts, USA) and R version 4.2.0 (http://www.R-project.org,The R Foundation).

## Results

### Participant characteristics

The demographic characteristics of the included participants are shown in **Table 1**. Compared with the controlgroup, the asthma group had a WWI index of 10.99(10.95,11.03), higher than 10.92(10.90,10.94)in the control group. 58.93% of the participants in the asthma group were females.

**Table 1:** Baselines characteristics of participants.

Characteristic	Non-asthma formers (*n*=38419)	Asthma formers (*n*=6061)	P-value
WWI Index	10.92 (10.90,10.94)	10.99 (10.95,11.03)	<0.0001
Gender (%)			<0.0001
Male	49.47 (48.94,50.00)	41.07 (39.45,42.72)	
Female	50.53 (50.00,51.06)	58.93 (57.28,60.55)	
Race (%)			<0.0001
Mexican American	14.15 (12.63,15.82)	10.57 (9.20,12.11)	
White	67.80 (65.56,69.95)	69.91 (67.40,72.31)	
Black	10.88 (9.76,12.12)	12.74 (11.33,14.28)	
Other Race	7.17 (6.52,7.89)	6.79 (5.90,7.80)	
Education Level (%)			0.0064
Less than high school	44.32 (42.90,45.75)	41.53 (39.53,43.56)	
High school	46.31 (45.03,47.59)	47.90 (45.90,49.91)	
More than high school	9.37 (8.36,10.49)	10.57 (8.73,12.75)	
Marital Status (%)			<0.0001
Cohabitation	65.10 (64.08,66.10)	58.41 (56.67,60.13)	
Solitude	34.90 (33.90,35.92)	41.59 (39.87,43.33)	
Alcohol (%)			0.433
Yes	63.03 (61.69,64.35)	64.13 (62.34,65.88)	
No	20.81 (19.66,22.00)	20.10 (18.68,21.60)	
Unclear	16.16 (15.37,16.99)	15.77 (14.45,17.19)	
High Blood Pressure (%)			<0.0001
Yes	29.61 (28.79,30.45)	34.96 (33.23,36.72)	
No	70.39 (69.55,71.21)	65.04 (63.28,66.77)	
Diabetes (%)			0.0002
Yes	8.47 (8.08,8.89)	10.20 (9.33,11.14)	
No	91.53 (91.11,91.92)	89.80 (88.86,90.67)	
Smoked (%)			<0.0001
Yes	45.15 (44.18,46.13)	49.95 (48.05,51.85)	
No	54.85 (53.87,55.82)	50.05 (48.15,51.95)	
Physical Activity (%)			0.7076
Never	28.89 (28.01,29.80)	28.24 (26.68,29.86)	
Moderate	31.70 (31.01,32.40)	31.81 (30.27,33.38)	
Vigorous	39.40 (38.47,40.35)	39.95 (38.05,41.88)	
Blood relatives had asthma (%)			<0.0001
Yes	17.79 (17.21,18.38)	40.90 (39.33,42.50)	
No	80.39 (79.77,81.01)	55.63 (53.96,57.28)	
Unclear	1.82 (1.65,2.00)	3.47 (2.90,4.15)	
Coronary Artery Disease			0.0108
Yes	3.25 (2.96,3.56)	4.15 (3.48,4.95)	
No	96.75 (96.44,97.04)	95.85 (95.05,96.52)	
Cancers			0.0006
Yes	9.28 (8.86,9.72)	11.30 (10.20,12.50)	
No	90.72 (90.28,91.14)	88.70 (87.50,89.80)	
PIR			<0.0001
<1.3	18.71 (17.78,19.68)	24.36 (22.78,26.01)	
≥1.3<3.5	33.69 (32.72,34.68)	31.94 (30.01,33.93)	
≥3.5	40.82 (39.37,42.28)	37.31 (35.00,39.68)	
Unclear	6.78 (6.27,7.33)	6.39 (5.55,7.35)	
Total Kcal (%)			0.2072
Lower	39.97 (39.25,40.71)	40.44 (38.72,42.17)	
Higher	47.15 (46.28,48.01)	45.71 (43.84,47.60)	
Unclear	12.88 (12.24,13.54)	13.85 (12.61,15.19)	
Total Sugar (%)			0.1271
Lower	38.22 (37.51,38.94)	37.07 (35.50,38.67)	
Higher	39.28 (38.47,40.10)	38.96 (37.38,40.57)	
Unclear	22.49 (21.84,23.16)	23.96 (22.57,25.42)	
Total Water (%)			0.0165
Lower	40.07 (39.30,40.85)	41.81 (39.98,43.65)	
Higher	47.05 (46.19,47.91)	44.34 (42.39,46.31)	
Unclear	12.88 (12.24,13.54)	13.85 (12.61,15.19)	
Total Sugar (%)			0.0884
Lower	39.37 (38.64,40.10)	40.29 (38.58,42.02)	
Higher	47.75 (46.93,48.57)	45.86 (44.02,47.71)	
Unclear	12.88 (12.24,13.54)	13.85 (12.61,15.19)	
Serum Cholesterol (%)			0.0001
Lower	46.71 (45.86,47.57)	50.19 (48.63,51.76)	
Higher	48.98 (48.08,49.88)	45.31 (43.77,46.85)	
Unclear	4.31 (4.00,4.64)	4.50 (3.84,5.27)	
Serum Glucose (%)			0.7931
Lower	48.48 (47.62,49.35)	48.69 (47.04,50.33)	
Higher	47.22 (46.32,48.13)	46.82 (45.12,48.52)	
Unclear	4.29 (3.98,4.62)	4.50 (3.84,5.27)	
Serum Uric Acid			0.5003
Lower	46.30 (45.58,47.02)	47.02 (45.45,48.59)	
Higher	49.39 (48.63,50.15)	48.45 (46.71,50.19)	
Unclear	4.31 (4.00,4.64)	4.54 (3.87,5.30)	

Data of continuous variables are shown as survey-weighted mean (95%CI), P value was calculated by survey-weighted linear regression. Data of categorical variables are shown as survey-weighted percentage (95%CI), P value was calculated by survey-weighted Chi-square test.

### Higher WWI index was associated with higher asthma prevalence

According to WWI index data, asthma prevalence is positively related to the WWI index. Likewise, the full-adjusted model (model 3) showed a stable relationship between WWI index and asthma(OR=1.15, 95% CI:1.11,1.20), indicating a 15% increase in asthma risk per unit increase. Additionally, we converted the WWI index from a continuous number to a categorical number (triple quantile) to analyze sensitivity. As shown in **Table 2**, Tertile 3 has a 29%higher risk of asthma occurrence(OR=1.29, 95% CI:1.19,1.40) than Tertile 1 in terms of WWI index (**Table 2**).

**Table 2:** Logistic regression analysis between WWI index with asthma prevalence.

Characteristic	Model 1 OR (95%CI)	Model 2 OR (95%CI)	Model 3 OR (95%CI)
WWI Index	1.09 (1.06,1.12)	1.26 (1.21,1.30)	1.15 (1.11,1.20)
Categories			
Tertile 1(7.59-10.38)	1	1	1
Tertile 2(10.38-11.71)	0.93 (0.87,1.00)	1.13 (1.05,1.21)	1.05 (0.97,1.13)
Tertile 3(11.71-15.70)	1.17 (1.10,1.25)	1.52 (1.41,1.64)	1.29 (1.19,1.40)

Model 1 was adjusted for no covariates;

Model 2 was adjusted for race, gender, age, marital status and education;

Model3 was adjusted for covariates in Model 2+diabetes, blood pressure, asthma, PIR, total water, total kcal, total sugar, smoked, physical activity, alcohol use,serum cholesterol, serum uric acid, coronary artery disease, cancers and serum glucose were adjusted.

### Subgroup analysis

To assess whether the correlation between the WWI index and asthma is robust,subgroup analyses were conducted. Results including:

- Male group (OR=1.19, 95% CI: 1.11,1.28),
- female group(OR=1.17, 95% CI: 1.11,1.23), age<40 years group(OR=1.07,95% CI: 1.01, 1.14),
- age 40-59 years group(OR=1.21, 95% CI:1.12, 1.30),
- age≥60 years group(OR=1.25,95% CI:1.16,1.35),
- white group(OR=1.19,95% CI:1.12,1.27),
- African American group(OR=1.18, 95% CI: 1.09, 1.28), others group(OR=1.27, 95% CI: 1.09, 1.47),
- hypertensive group(OR=1.22, 95% CI: 1.14,1.31),
- non-hypertensive group(OR=1.13, 95% CI: 1.07, 1.19),
- diabetic group(OR=1.36,95% CI: 1.22,1.52),
- non-diabetic group(OR=1.18, 95% CI: 1.13, 1.23),
- blood relative with asthma group(OR=1.21, 95% CI: 1.13, 1.29),
- blood relatives without asthma group(OR=1.13,95% CI: 1.07, 1.19) **(** **Table 3**).

**Table 3:** Subgroup analysis between WWI index with asthma prevalence.

Characteristic	Model 1 OR (95%CI)	Model 2 OR (95%CI)	Model 3 OR (95%CI)
Stratified by gender			
Male	0.94 (0.89,0.99)	1.28 (1.20,1.36)	1.19 (1.11,1.28)
Female	1.13 (1.09,1.18)	1.27 (1.22,1.33)	1.17 (1.11,1.23)
Stratified by age (years)			
20-39	1.03 (0.97,1.08)	1.11 (1.05,1.18)	1.07 (1.01,1.14)
40-59	1.38 (1.29,1.47)	1.38 (1.29,1.47)	1.21 (1.12,1.30)
60-85	1.32 (1.24,1.42)	1.33 (1.24,1.42)	1.25 (1.16,1.35)
Stratified by race			
Mexican American	1.13 (1.05,1.22)	1.12 (1.02,1.22)	1.07 (0.97,1.17)
White	1.09 (1.04,1.15)	1.31 (1.24,1.39)	1.19 (1.12,1.27)
Black	1.16 (1.09,1.24)	1.28 (1.19,1.38)	1.18 (1.09,1.28)
Other Race	1.20 (1.07,1.34)	1.36 (1.19,1.55)	1.27 (1.09,1.47)
Stratified by hypertension			
Yes	1.15 (1.09,1.22)	1.29 (1.22,1.38)	1.22 (1.14,1.31)
No	0.98 (0.94,1.03)	1.17 (1.12,1.23)	1.13 (1.07,1.19)
Stratified by diabetes			
Yes	1.31 (1.19,1.45)	1.47 (1.32,1.63)	1.36 (1.22,1.52)
No	1.04 (1.00,1.07)	1.25 (1.20,1.30)	1.18 (1.13,1.23)
Stratified by blood relative had asthma			
Yes	1.17 (1.11,1.23)	1.29 (1.21,1.37)	1.21 (1.13,1.29)
No	1.07 (1.03,1.12)	1.20 (1.14,1.26)	1.13 (1.07,1.19)
Unclear	0.99 (0.83,1.18)	1.26 (1.02,1.55)	1.18 (0.94,1.49)

Model 1=no covariates were adjusted.

Model 2=Model 1+race, gender, age, marital status and education were adjusted.

Mode 3=adjusted for all covariates except effect modifier.

### WWI ‘s dose response and threshold effect on asthma prevalence

**Figure 2:**
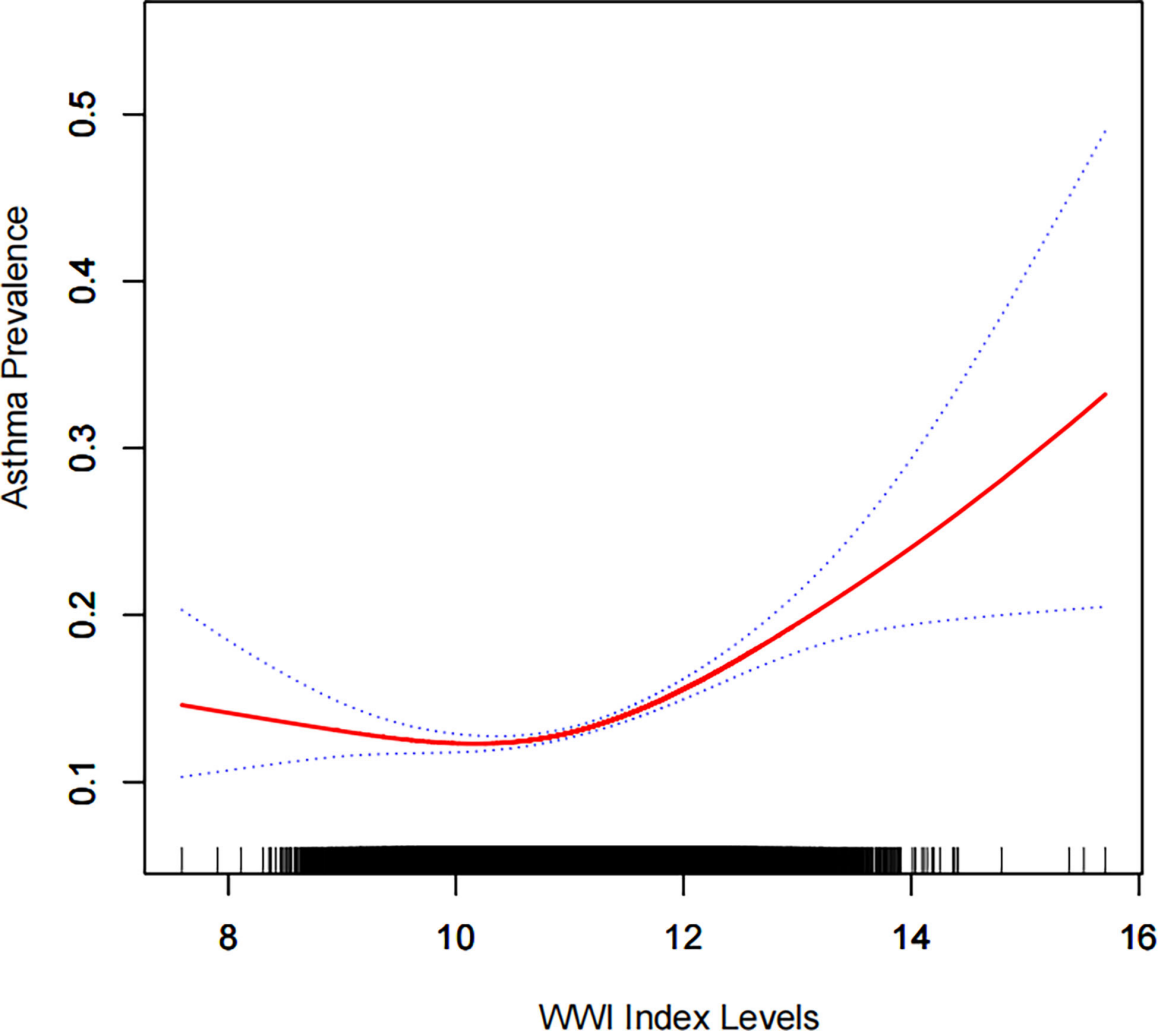
Density dose-response relationship between WWI index with asthma prevalence.The area between the upper and lower dashed lines is represented as 95% CI. Each point shows the magnitude of the WWI index and is connected to form a continuous line.Adjusted for all covariates except effect modifier.

We further explored the relationship between the WWI index and asthma using a generalized additive model and smoothed curve fitting. In **Figure 2**; **Table 4**, we found a nonlinear relationship between WWI index and asthma. Based on a two-segment linear regression model, the inflection point for the WWI is 10.53. (log-likelihood ratio test, p<0.001).

**Table 4:** Two-piecewise linear regression and logarithmic likelihood ratio test explained the threshold effect analysis of WWI index with asthma prevalence.

WWI Index	ULR TestOR (95%CI)	PLR TestOR (95%CI)	LRT testP value
<10.53	1.15 (1.11,1.20)	0.90 (0.81,0.99)	<0.0001
≥10.53		1.26 (1.20,1.33)	

ULR, univariate linear regression; PLR, piecewise linear regression; LRT, logarithmic likelihood ratio test, statistically significant: p<0.05.

### Elevated WWI index may delay age of first asthma onset

Fully adjusted model 3 exhibited a 3.89 year delay in asthma onset for every 1-unit increase in the WW index(β=3.89, 95% CI: 3.24, 4.54) (**Table 5**).

**Table 5:** Analysis between WWI index with onset age of asthma.

Characteristic	Model 1β (95%CI)	Model 2β (95%CI)	Model 3β (95%CI)
WWI Index	7.42 (6.87,7.98)	6.80 (6.22,7.39)	3.89 (3.24,4.54)

Model 1 was adjusted for no covariates;

Model 2 was adjusted for race, gender, marital status and education;

Model 3 was adjusted for covariates in Model 2+diabetes, blood pressure, asthma, PIR, total water, total kcal, total sugar, smoked, physical activity, alcohol use, serum cholesterol, serum uric acid, coronary artery disease, cancers and serum glucose were adjusted.

### WWI ‘s dose response and threshold effect on age of first asthma onset

**Figure 3:**
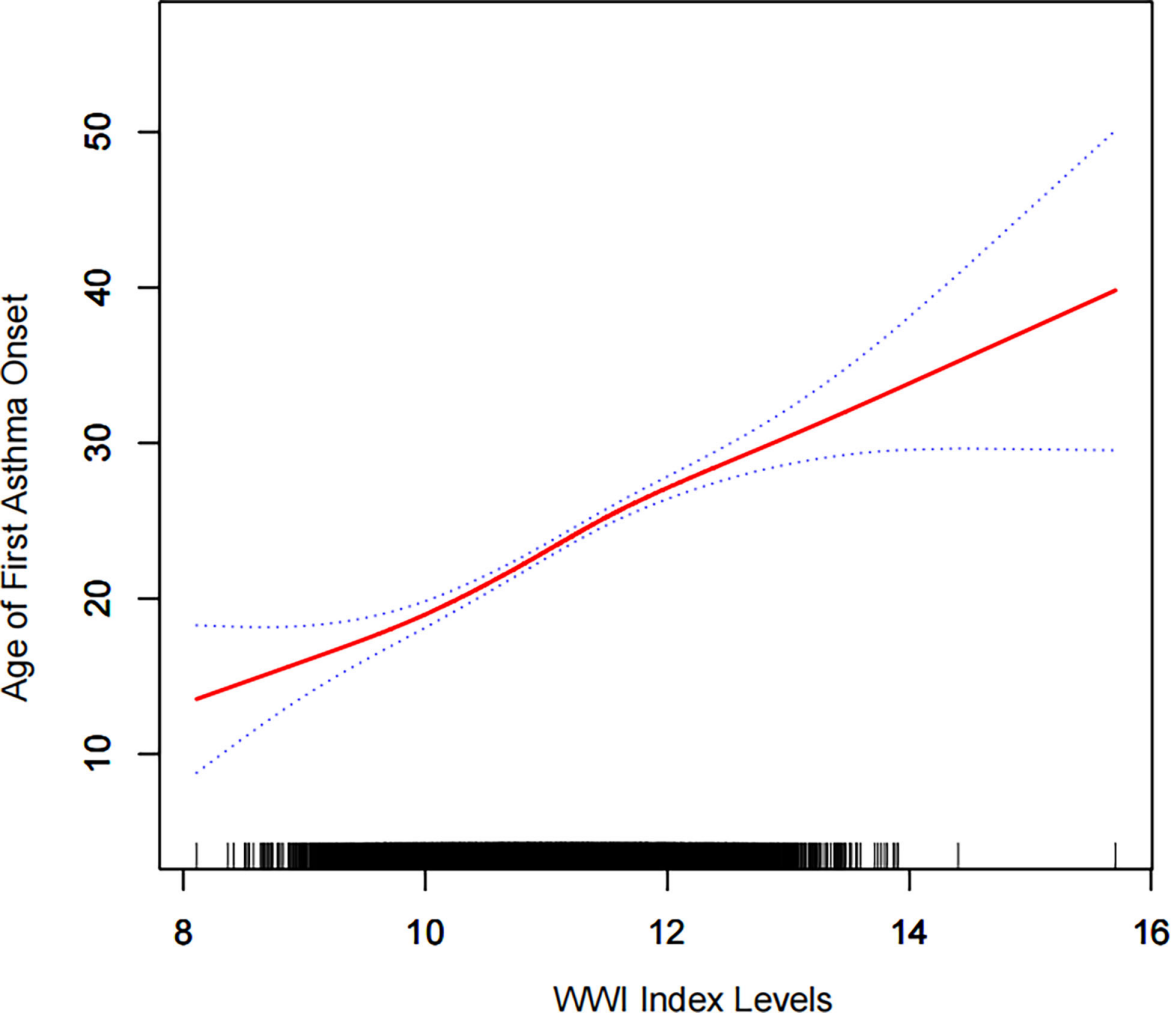
Density dose-response relationship between WWI index with onset age of prevalence.The area between the upper and lower dashed lines is represented as 95% CI. Each point shows the magnitude of the WWI index and is connected to form a continuous line.Adjusted for all covariates except effect modifier.

According to our results (**Figure 3**), we found a positive linear correlation between the WWI index and first asthma onset.

## Discussion

This study demonstrated a positive association between the WWI index and asthma prevalence among US adults. The WWI index increased by 1 unit was associated with a 15% increase in asthma prevalence. By using a generalized additive model and a smoothed curve fit, we were able to visualize the association between WWI and asthma prevalence clearly. Asthma prevalence and the WWI index did not have a linear correlation (**Figure 2**). The threshold and saturation effects showed that 10.53 was the most crucial inflection point.

Chronic diseases like asthma have placed increasingly huge burdens on health care costs, quality of life, and prevalence around the world (23). To prevent asthma, it is critical to practice primary prevention, especially targeting populations that are adapted to the WWI index may be the most effective. Accordingly, we conducted a sensitivity subgroup analysis and found out that almost all populations showed a positive association with asthma prevalence. This was excluding Mexican Americans and groups with unknown blood relatives with asthma. The WWI index has a significant positive association with asthma prevalence, indicating that the WWI index is widely used among asthmatic patients. In light of previous studies, we believe that our findings are accurate. First of all, when grouped by age, we found that older people had a higher prevalence of asthma. There was an increased prevalence of asthma with age, with a greater trend among men than among women (24). Asthma prevalence has also been found to be higher in older adults than in middle-aged adults, especially in men (25). We also found that high WWI prevalence among men was associated with higher asthma prevalence among women. In contrast, hypertension (26), diabetes mellitus (27, 28) had significantly higher asthma prevalence.

In epidemiological studies on asthma, phenotypes can be categorized according to the age at onset, the duration of the disease,and the clinical features of the disease (29). Asthma mortality and morbidity in older patients with asthma are higher than those in younger people (30). Asthma that develops late in life is susceptible to misdiagnosis and improper treatment, which can have profound negative consequences for the health of the patient (31, 32). It has also been shown in previous studies that abdominal obesity can lead to delayed-onset asthma, rather than early-onset asthma (33). A significant finding of this study is the correlation between the WWI index and the age of the first asthma attack. As a result of our findings, 3.89 years will be added to the age of asthma onset for each unit increase in the WWI index. WWI and age of first asthma onset were positively correlated even after smoothing curve fitting. It also implies that there is an increase in late-onset asthma. There is no report on this promising finding yet. This result needs to be confirmed by a large multicentre prospective study to further confirm its accuracy.

Asthma and obesity have associated mechanisms that have yet to be fully elucidated. It is possible to suggest several plausible relationships:

- There is an imbalance of adipokines secreted by adipose tissue,most commonly manifested as hyperleptinemia and low adiponectin levels (34), which results in chronic low-grade inflammation in the body. Airway hyperresponsiveness can be caused by adipokines released into the serum from inflamed adipose tissue (33). Asthma status was found to be positively correlated with serum leptin levels (35) and negatively correlated with serum adiponectin levels (36).
- Abdominal fat may mechanically reduce lung volume,especially when lying supine, by affecting the diaphragm and chest wall compliance. Airway hyperresponsiveness can develop when breathing low volume (37).

Furthermore, our study has several advantages. The NHANES 2001-2018 survey was conducted on a representative sample of the general U.S.population following a well-designed study protocol with extensive quality assurance and quality control procedures. Our results are reliable and can be applied to a broader range of individuals since they were adjusted for confounding covariates based on clinical understanding and previous studies. There are also limitations to our study. As a cross-sectional study, we were unable to establish a deterministic relation between the WWI index and asthma. Besides, the diagnosis of asthma was based on a questionnaire. Although previous studies have confirmed the acceptable accuracy of questionnaires (38, 39), recall bias remains. Furthermore, the database did not disclose detailed clinical variables such as medication history or asthma type classification, so further investigation is necessary. This study has many limitations, but its strength lies in its ability to reveal the relationship between the WWI index and asthma onset.

## Conclusion

This study showed an association between the modifiable risk factor WWI index and the prevalence of asthma and age at first asthma onset. A higher WWI index was associated with an increased prevalence of asthma and an earlier age of first asthma onset. Our findings suggest that weight control and a healthy lifestyle can reduce the occurrence of asthma, although the deterministic relation between the two cannot be clearly established, but is still of interest.

## Data availability statement

The datasets presented in this study can be found in online repositories. The names of the repository/repositories and accession number(s) can be found in the article/supplementary material.

## Ethics statement

The studies involving human participants were reviewed and approved by approved and reviewed by the National Center for Health Statistics Institutional Review Board(NCHS). The patients/participants provided their written informed consent to participate in this study.

## Author contributions

LY and YC: Conceptualization, methodology, software. MX, RL, JZ and ZH: Data curation, writing original draft. MC and GW: Writing-review & editing. All authors contributed to the article and approved the submitted version.
